# Effects of different supervised and structured physical exercise on the physical fitness trainability of children and adolescents: a meta-analysis and meta-regression: Physical fitness trainability in children and adolescents’ health

**DOI:** 10.1186/s12887-024-04929-2

**Published:** 2024-12-05

**Authors:** Carolina Dertzbocher Feil Pinho, Natália Carvalho Bagatini-PhD, Salime Donida Chedid Lisboa, Júlio Brugnara Mello, Giovani dos Santos Cunha

**Affiliations:** 1 Physiotherapy and Dance, Federal University of Rio Grande do Sul - School of Physical Education, 750, Felizardo Street – Jardim Botânico, Porto Alegre, 90690-200 Brazil; 2 Pontificia Universidad Católica de Valparaíso – Escuela de Educación Física – eFIDac Research Group, Valparaíso, Chile

**Keywords:** Muscular strength, Cardiorespiratory fitness, Muscular power, Maximal aerobic speed, Training

## Abstract

**Background:**

Physical fitness has been considered an important health indicator. Several factors can impact the increase in physical fitness in children and adolescents, including chronological age, sex and BMI, in addition to training variables such as weekly frequency, session and intervention duration, and types of exercises performed. To know the importance of variables that can impact physical fitness, it is important for health professionals to identify the most efficient way of prescribing physical exercises for children and adolescents. The aim is review and meta-analyses of the effects of supervised and structured physical exercise on the physical fitness trainability of children and adolescents.

**Methods:**

Relevant articles were searched in the PubMed, Cochrane Library, Embase and Scopus platform databases and selected based on the following criteria: children and adolescents aged between 7 and 17 years who performed any type of structured physical exercise compared to a control group without exercise and evaluating physical fitness (strength or muscular power, cardiorespiratory fitness (CRF) or speed. The results are reported in accordance with PRISMA 2020.

**Results:**

Eighty studies were included with a total of 5769 participants. Strength exercises (ES: 1.073; 95% CI, 0.612–1.533; *P* < 0.001; I2: 74%), concurrent (ES: 1.054; 95% CI, 0.255–1.853; *P* < 0.010; I2: 72%) and sports (ES: 0.573; 95% CI, 0.015 to 1.132; *P* < 0.044; I2: 34%) seem to be the most effective in increasing muscular strength. Aerobic activities (ES: 0.400; 95% CI, 0.258–0.542; *P* < 0.001; I^2^: 74%), sports (ES: 0.271; 95% IC, 0.148–0.394; *P* < 0.001; I^2^: 15), or HIIT (ES: 0.668; 95% IC, 0.333–1.003; *P* < 0.001; I^2^: 29%) resulted in increased CRF (ES: 0.514; 95% IC, 0.220–1.808; *P* < 0.001; I^2^: 66%). The practice of physical exercise increased muscular power (ES: 0.241; 95% CI, 0.053–0.429; *P* = 0.012; I^2^: 0%). The practice of HIIT impacts MAS gains (ES: 0.048; 95% CI, 0.050 − 0.026; *P* = 0.029; I^2^: 44%).

**Conclusion:**

Supervised and structured physical exercise can improve muscular strength (15–35%), CRF (5.4–8.5%), muscular power (5.6–11.8%), and MAS (5.4%) trainability in children and adolescents. Sex, BMI of the subjects and type of exercise performed (aerobic activities, exclusive to strength, HIIT or sports) should be considered when prescribing the exercise.

**Supplementary Information:**

The online version contains supplementary material available at 10.1186/s12887-024-04929-2.

## Background

Physical fitness is defined as a set of components related to the ability to perform physical activities [1]. The components include musculoskeletal fitness (MF – composed of muscular strength, power, endurance, and flexibility), cardiorespiratory fitness (CRF), motor fitness (speed and agility), and body composition (muscular mass, body fat, and bone mass), which are associated with health indicators and physical-sports performance [1]. In the pediatric population, evidence shows that individuals with high levels of physical fitness also have better musculoskeletal and metabolic health, aerobic endurance, motor coordination, neural development, social well-being, and favorable body composition (lower percentage of fat and greater muscle mass) and have a lower risk of early onset of chronic diseases [2–7].

To increase physical fitness, it is recommended that young people practice 60 min of physical activity of moderate-vigorous intensity daily, including strength activities at least 3 times a week for muscle and bone health [8]. To potentiate the effects, supervised and structured physical exercise is recommended [8, 9], considering the principles of physical-sports training (volume, intensity, weekly frequency, session duration, intervention duration and type of physical exercise) [10]. Despite the evidence demonstrating the importance of physical exercise for health [11, 12], it is estimated that factors such as advancing chronological age, sex physiological differences, and different body compositions seem to influence the levels of physical fitness. Recent meta-analyses with 52 studies and more than 22,000 participants aged between three and 18 years show that physical exercise practices decline at age 9 for boys and girls becoming sedentary and at high risk for metabolic diseases in adult life. In this way, it is important to know how these principles can influence the physical fitness and health of children and adolescents [13], since this is a period of body and mental transition, and these factors can impact physical fitness gains.

Despite the much evidence on the positive effects of physical fitness on the health and performance of children and adolescents [14–17], there is still an important practical issue regarding the prescription of training for this population with a focus on health. Several reviews summarize training effects [15, 18, 19], but there is still no consensus on which training methodology, weekly frequency, volume, and intensity is most effective for each component (or globally) of physical fitness in young people. For example, Miller et al. [2010] indicate that when correctly prescribed and considering the training variables, children and adolescents who practice strength training can develop muscular, neural, and biomechanical strength [20, 21]. On the other hand, Enríquez-Del-Castillo et al. [22] reported that moderate-intensity exercise can improve muscular strength, CRF and motor ability. However, high-intensity interval training (HIIT) and sports participation [23–25] are dynamic and pleasure activities for the pediatric population, and studies have presented positive effects on physical fitness components and cardiometabolic parameters. In this regard, there are many ways to improve physical fitness, but it is still rare which methodologies are most suitable for child population and physical fitness improvements, considering the principles of training, whereas there is a relationship between physical fitness, health, and performance.

In this way, exercise is considered a polypill with low cost, is relatively free of adverse effects and can prevent and combat the early onset of chronic diseases resulting from a sedentary lifestyle [26]. However, there is still little evidence on how children and adolescents respond to each training methodology, and which would be most appropriate for improving the components of physical fitness for healthy growth in both cardiorespiratory and muscular strength. It is important to note that all systematic reviews focused on specific outcomes such as MF [4, 27, 28], CRF [29, 30] or health [28, 29, 31], and to our knowledge, no review with meta-analysis presents methodologies for more than one component (MF (strength and power), CRF and maximum speed) with different exercise training methods. Additionally, there is much evidence about the effects of different physical exercise strategies on physical fitness health in children and adolescents, which makes it difficult to identify the best way to prescribe physical exercise for the pediatric population. Furthermore, it is important that health professionals identify the most efficient way of prescribing physical exercises to improve physical fitness and health, with the aim of making children and adolescents more active.

Therefore, the aim of this review and meta-analysis is to explore the effects of different supervised and structured physical exercise prescriptions on the physical fitness trainability of children and adolescents. Furthermore, we identified the contribution of the principles of physical sports training (volume, intensity, weekly frequency, session duration, intervention duration and type of physical exercise) in different subjects (age, sex, and BMI).

## Methods

This systematic review was conducted using a predetermined protocol established according to the recommendations of the Cochrane Handbook [32] and was previously registered at the international prospective register of systematic reviews (PROSPERO number registration CRD42021266583) and available at https://www.crd.york.ac.uk/prospero/display_record.php?ID=CRD42021266573. The results are reported in accordance with the PRISMA 2020 (PRISMA Statement) [33] (Additional file 1).

### Eligibility criteria

Original articles published in journals or “ahead of print” were eligible for consideration. The search included no time or language restrictions. Only eligible full texts in English, Portuguese, or Spanish were considered for review. Studies were considered eligible for inclusion if they provided relevant information on PICOS strategy (participants, interventions, comparators, outcomes, and study design) and met the following inclusion criteria: (1) participants: children and adolescents aged 7–17 years; (2) interventions: all types of structured and supervised physical activity intervention; (3) comparator: control groups that did not receive exercise intervention; (4) outcome: at least one evaluation of physical fitness (muscular strength, cardiorespiratory fitness, muscular power and speed); (5) study design: clinical trials (randomized or not) with pre- and post-measures. The exclusion criteria were as follows: (1) trials contained adults; (2) interventions targeted specific groups of children, such as those with mental illness or disability; (3) young athletes; (4) the physical activity involved using smart devices, such as mobile phones or video games; (5) the control group performed an intervention (physical education + exercise); and (6) duplicate publications or sub studies of included trials.

### Information source

A search for articles was performed in September 2021 and updated in July 2022 using the following electronic databases: MEDLINE (accessed by PubMed), Cochrane Library, Embase, and Scopus. The abstracts of these articles were checked for the relevant inclusion criteria, and if necessary, the full text was investigated.

### Search strategy

The search terms included “children”, “adolescents”, “strength training”, “aerobic exercise”, “plyometric exercise”, “sport”, “muscular fitness”, “cardiorespiratory fitness”, “sprint” and related entry terms (Additional file 2). The records identified were PubMed (*n* = 3391), Cochrane Library (*n* = 5973), Embase (*n* = 956) and Scopus (*n* = 1516).

### Selection process and data extraction process

The titles and abstracts of the retrieved articles were independently evaluated by 3 investigators (C.D.F.P., N.C.B, and S.D.C.L.). One reviewer (C.D.F.P.) independently screened all studies, and two reviewers independently screened half of the studies (N.C.B. and S.D.C.L.), both in the selection of studies and in the data extraction. The reviewers were not blinded to the authors, institutions, or manuscript journals. Abstracts that did not provide enough information regarding the inclusion and exclusion criteria were retrieved for full-text evaluation. Reviewers independently evaluated full-text articles and determined study eligibility. Disagreements were resolved by consensus, and if any disagreement persisted, it was resolved with a third reviewer (C.D.F.P.). To avoid possible double counting of participants included in more than one report by the same authors or working groups, the participant recruitment periods were evaluated, and if necessary, authors were contacted for clarification. The corresponding author was contacted as needed to obtain data not included in the published full-text report. A standardized form was used for data extraction, which was presented to the reviewers, and they should describe the author and year of the article. Subsequently, the total number of participants and their characteristics (sex, age, body mass, height, body fat (%BF), free-fat mass (FFM) and BMI) were described when they had this information. The characteristics of the interventions should be described with the type of exercise performed, duration of follow-up, duration of session, intensity, and weekly frequency. Finally, the results should be extracted for the exercise and control groups. The outcomes of interest extracted were muscular strength (kg), cardiorespiratory fitness (ml.kg^− 1^.min^− 1^), muscular power (*countermovement jump* – CMJ) and maximal aerobic speed (MAS – km.h^− 1^). Studies that presented other units of measurement were selected but not described in the statistical analysis due to the choice of using gold standard tests to present the results. The same 3 reviewers (C.D.F.P., N.C.B. and S.D.C.L.) who screened the articles and conducted data extraction in an independent way. Disagreements were resolved by consensus or by the reviewer (C.D.F.P.).

### Assessment of methodological quality

The same two reviewers (C.D.F.P. and S.D.C.L.) who independently performed the study selection and data extraction, and a third reviewer (J.B.M) assessed the methodological quality of the included studies. For this, the tool for the assessment of study quality and reporting in exercise training studies was proposed by Smart et al. (2015) [34]. As such, the assessment included the following items: (a) specified eligibility criteria; (b) specified randomization; (c) allocation concealment, that is, patients are unaware of which group they would be allocated; (d) similar groups at baseline, with no significant difference after randomization; (e) blinding of outcome evaluators; (f) outcome measures evaluated in at least 85% of patients; (g) intention-to-treat analysis; (h) reporting of statistical comparison between groups; (i) point measures and measures of variability for all reported outcomes; (j) monitoring of activities in the control group; (k) whether relative exercise intensity remained constant; and (l) relative volume and energy expenditure of the exercise. The quality score of the papers was based on terciles, where 0 to 5 points was considered low quality, 6 to 10 points was considered medium quality, and 11 to 15 points was considered high quality. This instrument was chosen because it is specific for interventions with physical exercise.

### Statistical analysis

The pooled effect estimates were computed from the change scores between the baseline and the end of intervention, their standard deviations (SDs), and the number of participants in each group. Data from intention-to-treat analyses were entered whenever available in the included studies. The authors were contacted through e-mails for unreported data, and if no answer returned or if the data requested were not available, the studies were excluded. The results are presented as standardized mean differences (a measure of effect, recommended to be used when the study reports efficacy of an intervention on continuous measurements, especially in cases of different methods of measurement), and calculations were performed using random effects models. Statistical heterogeneity of treatment effects among studies was evaluated by the Cochran Q test and the I^2^ inconsistency test; values > 50% indicated high heterogeneity [32]. Forest plots were generated to present the pooled effects and standardized mean differences with 95% confidence intervals (CIs). In addition, sensitivity analyses were conducted to investigate the possible influence of sex (male, female or both sexes) and type of training (strength training, aerobic training, HIIT, concurrent training, and sports) on adaptations achieved. Meta-regression analyses were performed to investigate potential moderators: mean age (years), BMI (kg.m^-2^), follow-up duration (weeks), weekly frequency (number of sessions per week), and session duration (min). Values of *p* < 0.05 were considered statistically significant. All analyses were performed using OpenMeta Analyst Software, version 10.10 [35].

To enhance the results, sensitivity analyses were performed excluding each study separately to analyze the influence of each study on the overall results. The percent change (Δ%) was calculated for each study to assess the magnitude of effects using the following equation:

$${\rm{\Delta \% }}\,{\rm{ = }}\,\left( {{\rm{Mpost}} - {\rm{Mpre}}} \right)\,{\rm{Mpre}}\,{\rm{ \times }}\,{\rm{100}}$$

where Mpost represents the average value after long-term training and Mpre the average baseline value.

Effect size (ES) was calculated to account for standardized long-term training effects on outcome variables (e.g., muscular strength and power, CRF, and MAS). The ES was calculated using Cohen’s d [36, 37], dividing the raw ES (difference in means) by the pooled standard deviations:

$$ {\rm{ES}}\,{\rm{ = }}\,\left( {{\rm{Cohen's}}\,{\rm{d}}} \right)\,{\rm{ = }}\,\left( {{\rm{M1}} - {\rm{M2}}} \right){\rm{/SD}}\,{\rm{grouped}} $$

Values for ES were defined as trivial (< 0.2), small (0.2–0.6), moderate (0.6–1.2), large (1.2–2.0) and very large > 2 [38].

## Results

### Study selection

**Fig. 1:**
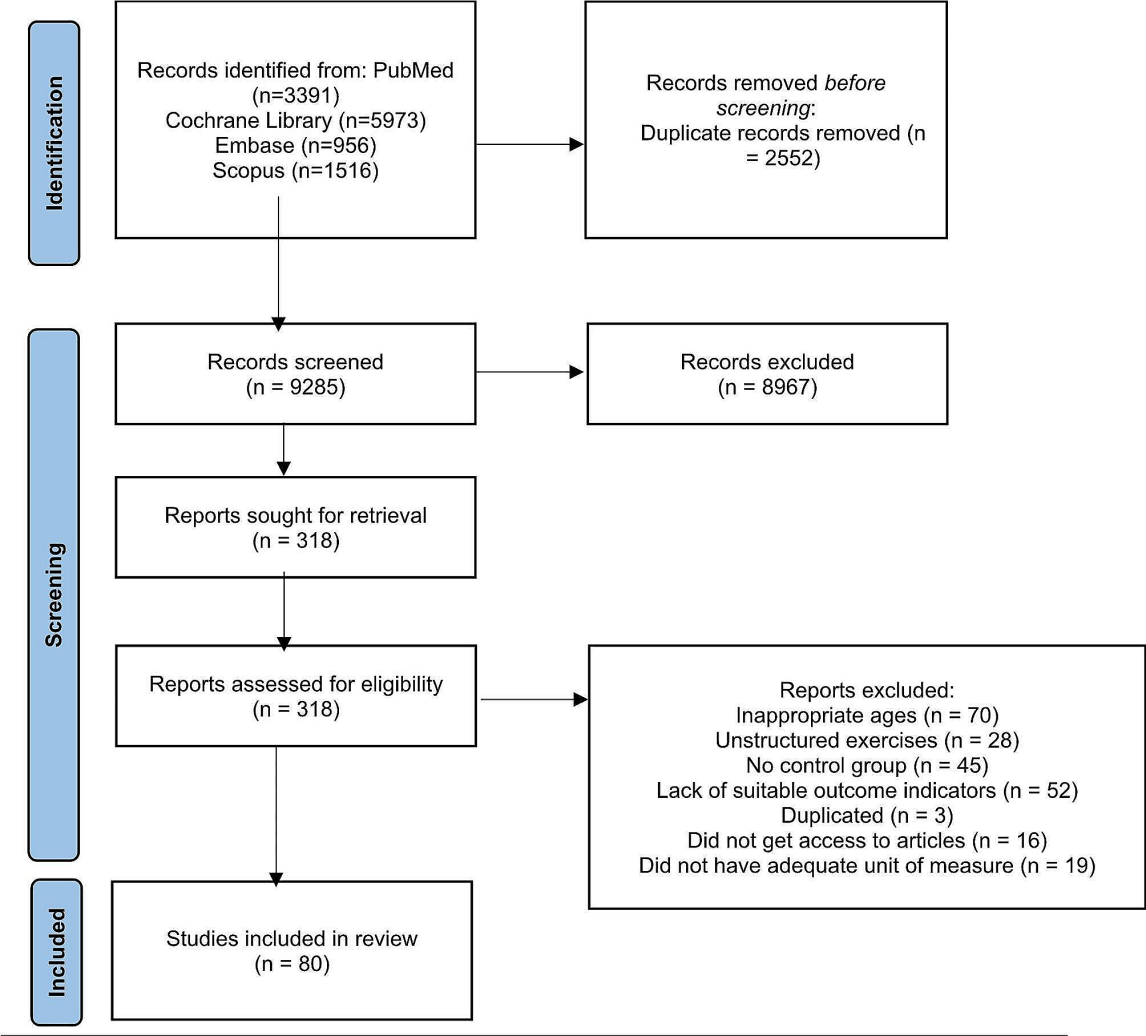
Flowchart of the number of articles retrieved during the literature search and study selection

The search of databases yielded a total of 11,837 citations. After adjusting for duplicates, 9285 studies remained. Of these, 8967 were discarded because after reviewing the titles and abstracts, it appeared that these papers did not meet the eligibility criteria. The full texts of the remaining 318 citations were examined in more detail. It appeared that 238 studies did not meet the inclusion criteria. Thus, 80 studies [39–105] met the inclusion criteria and were included in the quantitative analysis (Fig. 1). No additional studies were identified by checking the references of the included papers. No relevant unpublished studies were obtained.

### Study characteristics

In total, 5769 participants were included in the meta-analysis. Among these, 2894 were included in the exercise group, and 2875 participants were included in the control group. Of these, 65% were studies with both sexes, 22.5% were only boys and 12.5% were only girls.

Only quantitative studies that presented “MF – muscular strength” included 297 children or adolescents in the exercise group, while 241 were included in the control group. A total of 43.75% of the studies analyzed both sexes, 43.75% analyzed only boys, and 12.5% analyzed only girls. In 75% of studies, participants were classified by normal BMI, and 25% were overweight or obese children. The selected studies with strength training intervention were carried out during a period between 8 and 20 weeks, with a weekly frequency of 2 to 3 times a week. Regarding the duration of the session, the average of the sessions was 30 to 60 min, and the intensity was controlled by the rating of perceived exertion (BORG scale 15–18), percentage of 1RM (62–97%), percentage of 10RM (50–100%) and 6-20RM. The selected studies with concurrent training intervention were carried out during a period between 8 and 12 weeks, with a weekly frequency of 3 times a week. Regarding the duration of the session, the average of the sessions was 60–70 min, and the intensity was low-moderate for aerobic exercises, which could be controlled by maximum heart rate (70–95% HR_max_) or VO_2peak_ (50–85%), while strength training was performed with an intensity of 8-10RM. Interventions with sports practice were carried out for 12 or 16 weeks, with a weekly frequency of 3 times a week, the duration of the session was 50–60 min and the intensity was above 90% of HR_max_.

For the CRF outcome, a total of 2273 children or adolescents were included in the exercise group, while 2340 were included in the control group. A total of 76.7% of the studies analyzed both sexes, 16.3% analyzed only boys, and 7.0% analyzed only girls. In 44.2% of studies, participants were classified by normal BMI, 34.9% were overweight or obese children, and 20.9% of the studies did not provide this information. The studies selected were classified according to moderate-intensity aerobic activities (treadmill, bicycle, running or jumping rope) [40, 42, 48, 52, 55–85, 88–93, 102, 105, 106], which had an intervention duration between 7 and 18 weeks and were performed 3 and 4 times a week. The duration of the sessions ranged from 30 to 63 min, and the intensity varied between 50 and 80% VO_2peak_, 70–95% HR_max_ and > 70% of the maximum aerobic speed. Interventions with concurrent training were carried out between 8 and 12 weeks, with a weekly frequency of 2–4 times a week. The duration of the session was between 20 and 90 min, and the intensity was controlled by maximum aerobic speed (> 70%) and 50–85% of VO_2peak_, while for strength training, the intensity was between 70 and 80% 1RM. Sports practices were carried out between 8 weeks and up to 2 years of intervention, with activities being carried out three times a week to daily. Session durations ranged from 20 to 80 min, and the intensity was controlled to be > 78% HR_max_. Finally, studies that performed a HIIT intervention lasted from 7 to 12 weeks and were practiced between 1 and 3 times a week. Sessions lasted from 16 to 60 min, with intensity controlled by maximal aerobic velocity (> 100%), HR_max_ (85–95%) and VO_2peak_ velocity (100%).

For the “MF – muscular power” outcome, 220 children or adolescents were included in the exercise group, while 225 were included in the control group. A total of 46.2% of the studies analyzed both sexes, 23.1% analyzed only boys, and 30.7% analyzed only girls. In 53.8% of studies, participants were classified by normal BMI, 15.4% were overweight or obese children, and 30.8% of the studies did not provide this information. The selected studies analyzed strength training lasting between 8 and 10 weeks, and activities were performed two or three times a week [39, 56, 83, 86, 94–102]. The sessions lasted from 45 to 90 min, and intensity was controlled based on maximum repetitions (1RM, 6 or 10RM) or maximum aerobic speed. The study that carried out concurrent training (plyometrics on the trampoline + strength training) lasted four weeks with 45-minute sessions and a weekly frequency of three times a week. The studies that performed the HIIT intervention had an intervention duration between 7 and 12 weeks and a weekly frequency of three times a week. The sessions lasted 30 min, and the intensity was controlled based on HR_max_ or VO_2peak_ velocity.

For the MAS outcome, 104 children or adolescents were included in the exercise group, while 71 were included in the control group. A total of 83.3% of the studies analyzed both sexes, 16.7% analyzed only girls, and no study analyzed only boys. In 33.3% of studies, participants were classified by normal BMI, 33.3% were overweight or obese children, and 33.3% of the studies did not provide this information. All studies performed a HIIT intervention lasting between six and 12 weeks and weekly frequency ranging from two to three times a week [58, 71, 78, 103, 104, 107]. The sessions lasted from six minutes to one hour, and the intensity was measured through the maximum aerobic speed (> 70%).

### Methodological quality of the included trials

The risk of bias was determined using the TESTEX scale. Overall, the studies included in this review were of medium quality (between 5 and 10 points on the TESTEX scale) (Additional file 3). Studies with “MF – muscular strength” [39, 40, 42–54, 106] outcomes were mostly of medium quality (9 studies scored between 7 and 10). Only three were rated high-quality. Studies with VO_2max_ [40, 42, 48, 52, 55–85, 88–93, 102, 105, 106] results were mostly of medium quality. Only seven were classified as high quality (scored between 11 and 14), and 35 were classified as medium quality (scored between 7 and 10). Studies with “MF – muscular power” [39, 56, 83, 86, 94–102] results were mostly of medium quality (10 studies scored between 6 and 10). In this case, no study was of low quality. All studies with maximal aerobic speed [58, 71, 78, 103, 104, 107] results were of average quality (6 studies scored between 6 and 10).

### Effectiveness of supervised and structured physical exercise on muscular strength

**Fig. 2:**
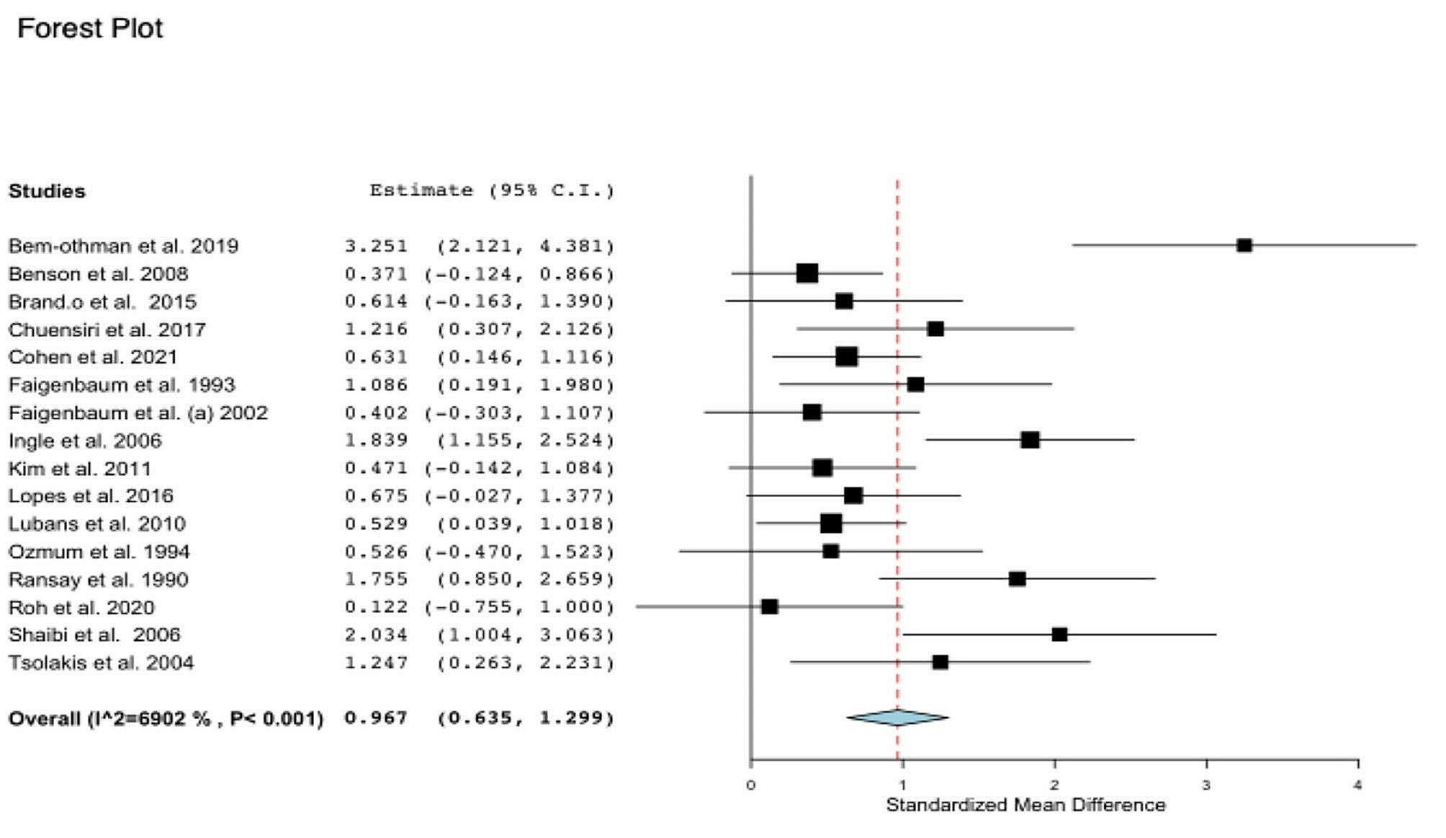
Standardized mean differences of “MF – muscular strength” in the intervention versus control groups (no intervention) Note: CI: confidence interval

Data concerning MF were available from 16 studies, which compared exercise training interventions versus control groups in a total of 455 participants aged between 8 and 15 years old (Fig. 2). Regarding the intervention, 10 studies performed a strength training intervention, three studies performed concurrent training, and three studies performed sports training. Muscular strength was measured using the repetition maximum (RM) (1RM, 6RM or 10RM) of upper-lower body strength with a bench press, elbow flexors, squats, leg press or knee extension. Exercise intervention was associated with a large effect size with improved muscular strength compared with no intervention (ES: 0.967; 95% CI, 0.635 to 1.299; *P* < 0.001; I^2^: 69%).

Regarding the subgroup analysis, the sex of participants can impact the magnitude of gains in strength. According to the seven studies that analyzed only boys, physical exercise can impact muscular strength in a large effect size (ES: 1.67; 95% IC, 0.911 to 2.440; *P* < 0.001; I^2^: 76%). In girls (two studies) and studies with both sexes (seven studies), muscular strength gain was considered to have a moderate effect size (ES: 0.55; 95% CI, 0.097 to 1.021; *P* < 0.018; I^2^: 0) (ES: 0.59; 95% CI, 0.364 to 0.816; *P* < 0.001; I^2^: 0%), respectively.

Additionally, regarding the subgroup analysis considering the type of training, studies with strength training intervention (ES: 1.073; 95% CI, 0.612 to 1.533; *P* < 0.001; I2: 74%) resulted in a large effect size in the magnitude of muscular strength, with a change of 31.32%, as well as concurrent training (strength + aerobic) (ES: 1.054; 95% CI, 0.255 to 1.853; *P* < 0.010; I2: 72%), with a change of 34.49%. On the other hand, studies with sports (ES: 0.573; 95% CI, 0.015 to 1.132; *P* < 0.044; I2: 34%) resulted in a moderate effect size with a change of 15.16%.

According to the results of the meta-regression analysis, the longer the duration of the session, the greater the increases in muscular strength (β: 0.041; 95% CI; 0.014 to 0.069; *P* = 0.003). However, mean age, BMI, weekly frequency, and follow-up (weeks) were not associated with improvements in strength (Table 1).

**Table 1:** Meta-regression of moderators of the physical fitness

Outcome/moderator	Number of study estimates	β	95% IC	*P* value
*Muscular Strength*				
Mean age	16	-0.071	-0.251 to 0.110	0.44
BMI	16	-0.015	-0.129 to 0.099	0.79
Weekly frequency	16	0.042	-0.407 to 0.490	0.85
Session duration	16	0.041	0.014 to 0.069	**0.03**
Follow-up duration (weeks)	16	0.030	-0.060 to 0.120	0.50
*Aerobic Training*				
Mean age	43	0.074	-0.008 to 0.156	0.78
BMI	43	0.048	0.016 to 0.081	**0.04**
Weekly frequency	43	0.016	-0.115 to 0.146	0.81
Session duration	43	0.003	-0.002 to 0.007	0.26
Follow-up duration (weeks)	43	-0.005	-0.014 to 0.004	0.27
*Muscular Power*				
Mean age	12	-0.003	-0.074 to 0.067	0.92
BMI	12	-0.020	-0.113 to 0.047	0.67
Weekly frequency	12	0.223	-0.167 to 0.614	0.26
Session duration	12	0.002	-0.009 to 0.013	0.70
Follow-up duration (weeks)	12	-0.035	-0.139 to 0.069	0.50
*Maximal Aerobic Speed*	6			
Mean age	6	0.118	0.003 to 0.232	**0.04**
BMI	6	0.172	0.052 to 0.292	**0.05**
Weekly frequency	6	0.325	-0.132 to 0.782	0.16
Session duration	6	-0.001	-0.017 to 0.015	0.86
Follow-up duration (weeks)	6	0.102	-0.030 to 0.234	0.12

Bold denotes statistically significant difference (*p* < 0.05). Abbreviations: BMI: body mass index; CI: confidence interval

### Effectiveness of supervised and structured physical exercise on cardiorespiratory fitness

**Fig. 3:**
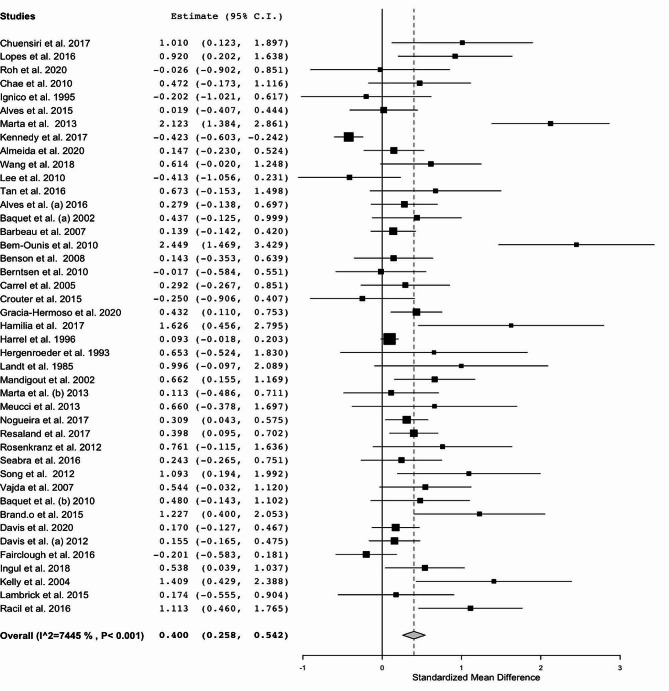
Standardized mean differences in CRF between the intervention and control groups (no intervention) Note: CI: confidence interval

Data concerning CRF were available from 43 studies, which compared exercise training interventions versus control groups in a total of 4363 participants aged between 7 and 17 years old (Fig. 3). Regarding the intervention, ten studies performed aerobic training, five studies performed a strength training intervention, seven studies performed concurrent training, fourteen studies performed sports training and five studies performed HIIT. CRF was measured using maximum or peak oxygen consumption (VO_2max_ or VO_2peak_). The exercise group was associated with an improvement in CRF with a change of 6.49% in VO_2max_ compared with controls who did not exercise (ES: 0.400; 95% CI, 0.258 to 0.542; *P* < 0.001; I^2^: 74%).

Regarding the subgroup analysis, the sex of participants can impact the magnitude of gains in CRF. According to the seven studies that analyzed only boys (ES: 0.378; 95% CI, 0.169 to 0.586; *P* < 0.001; I^2^: 0%) and 33 studies with both sexes (ES: 0.395; 95% CI, 0.222 to 0.567; *P* < 0.001; I^2^: 78%), physical exercise can impact a moderate effect size. However, three studies analyzed only girls, and exercise was not associated with an increase in the magnitude of CRF (ES: 0.669; 95% IC, -0.024 to 1.361; *P* = 0.059; I^2^: 79%).

Furthermore, in subgroup analysis of type of training, studies with intervention with aerobic training (*n* = 11) showed a significant increase in magnitude of CRF (ES: 0.514; 95% IC, 0.220 to 1.808; *P* < 0.001; I^2^: 66%), resulting in a moderate effect size with a change of 8.45%. Studies with HIIT (*n* = 6) training also show a significant difference in CRF (ES: 0.668; 95% CI, 0.333 to 1.003; *P* < 0.001; I^2^: 29%), resulting in a moderate effect size with a change of 8.20%. In addition, studies with sports (ball games, soccer, taekwondo, dance, and *capoeira*) (*n* = 14) showed a significant increase in the magnitude of CRF (ES: 0.271; 95% CI, 0.148 to 0.394; *P* < 0.001; I^2^: 15%), resulting in a small effect size with a change of 5.40%. However, exclusive strength training (*n* = 5) (ES: 0.440; 95% IC, -0.460 to 1.341; *P* = 0.338; I^2^: 93%) and concurrent training (strength + aerobic) (*n* = 8) (ES: 0.360; 95% IC, -0.054 to 0.775; *P* = 0.089; I^2^: 77%) did not show significant increases in CRF adaptations.

The greater the BMI of the participants was, the greater the magnitude of improvement (β: 0.048; 95% CI; 0.048 to 0.081; *P* = 0.004). However, mean age, weekly frequency, duration of session, and follow-up (weeks) were not associated with improvements in this outcome (Table 1).

### Effectiveness of supervised and structured physical exercise on muscular power

**Fig. 4:**
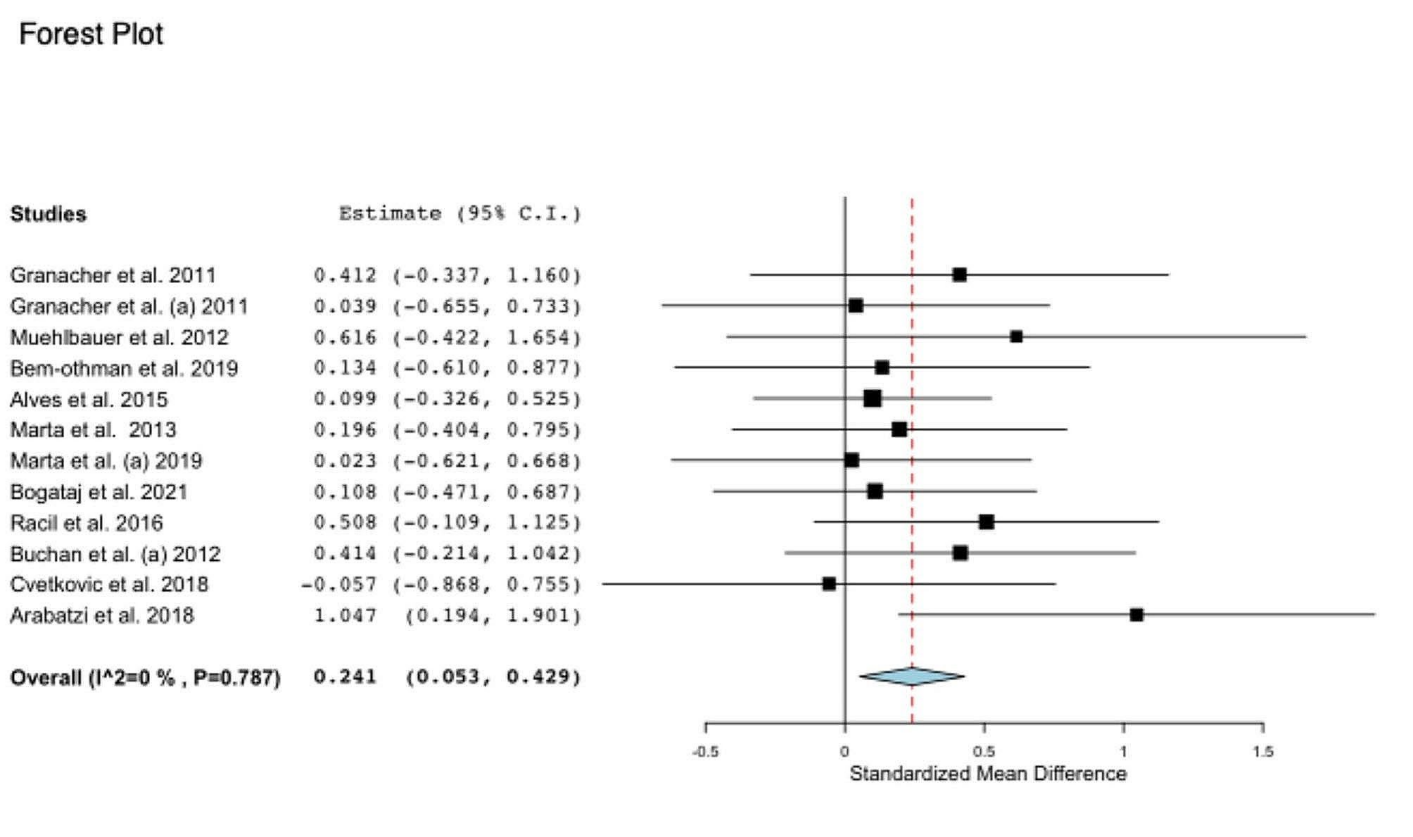
Standardized mean differences of “MF – muscular power” in intervention versus control (no intervention) Note: CI: confidence interval

Data regarding muscular power variables were available from 12 studies, which compared exercise training interventions versus control groups in a total of 445 participants aged between 8 and 17 years old (Fig. 4). Regarding the intervention, seven studies performed a strength training intervention, one study performed concurrent training, three studies performed HIIT, and two studies performed sports training. In all studies, muscular power was measured using CMJ. The exercise intervention was associated with a small improvement in jumping height, with a change of 9.27% (ES: 0.241; 95% CI, 0.053 to 0.429; *P* = 0.012; I^2^: 0%) compared to the control group.

Regarding the subgroup analysis considering sex, studies with intervention with boys and girls in the same group (*n* = 6) (ES: 0.292; 95% IC, 0.050 to 0.535; *P* = 0.018; I^2^: 0%) had a small effect size and increased the magnitude of jumping height. However, studies with only boys (*n* = 3) (ES: 0.037; 95% IC, -0.381 to 0.455; *P* = 0.213; I^2^: 0%) or only girls (*n* = 4) (ES: 0.295; 95% IC, -0.127 to 0.717; *P* = 0.171; I^2^: 0%) were not associated with an increase in the magnitude of jump height.

Additionally, in subgroup analyses of type of training, studies did not show a significant increase in jumping height with only strength training (*n* = 7) (ES: 0.189; 95% IC, -0.102 to 0.481; *P* = 0.203; I^2^: 0%) with a change of 7.66%, concurrent training (*n* = 2) (ES: 0.498; 95% IC, -0.419 to 1.416; *P* = 0.287; I^2^: 73%) with a change of 11.76%, and HIIT (*n* = 3) (ES: 0.332; 95% IC, -0.018 to 0.682; *P* = 0.063; I^2^: 0%) with a change of 5.62%. Sports intervention could not enter the analysis due to the low number of studies (*n* = 2). Mean age, BMI, weekly frequency, duration of session and follow-up (weeks) were not associated with improvements in jumping height (Table 1).

### Effectiveness of supervised exercise training in maximal aerobic speed

**Fig. 5:**
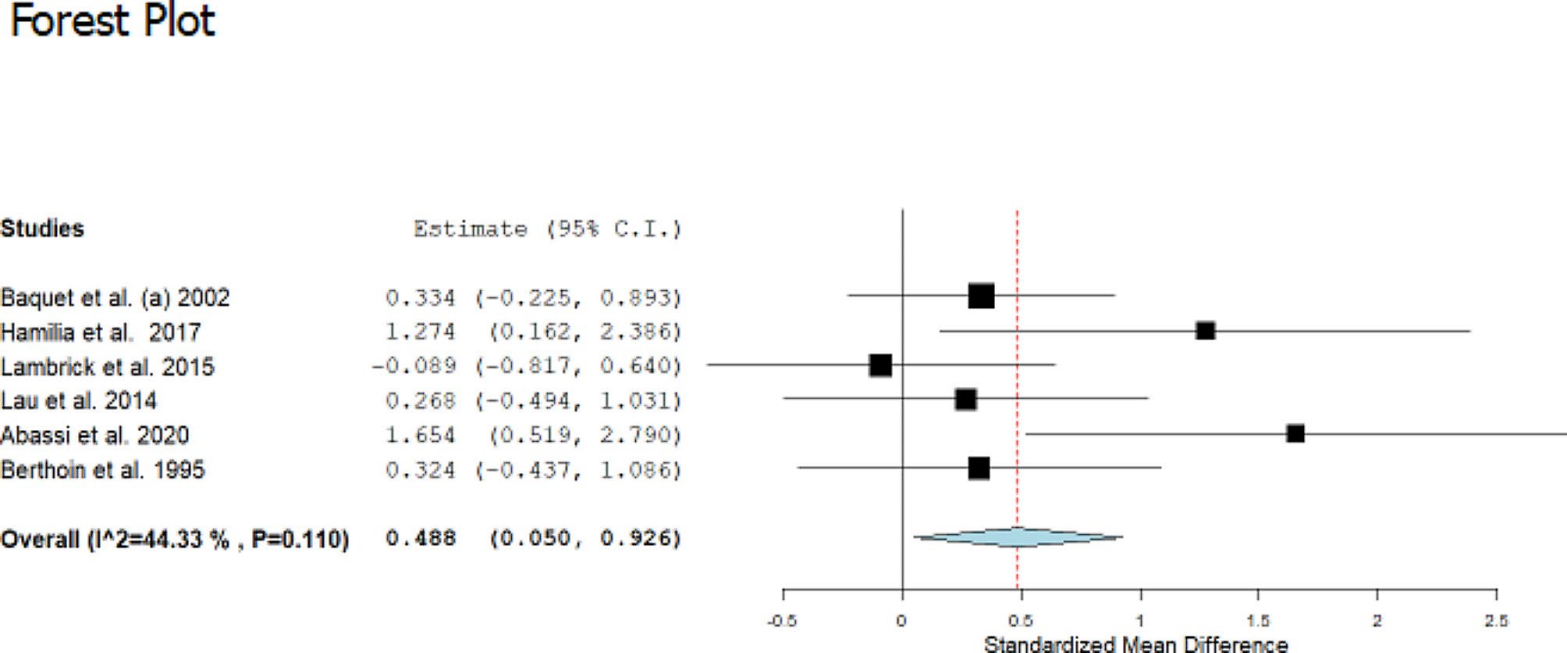
Standardized mean differences in maximal aerobic speed in the intervention versus the control (no intervention) Note: CI: confidence interval

Data regarding maximal aerobic speed variables were available from 6 studies, which compared exercise training interventions versus control groups in a total of 175 participants aged between 9 and 17 years old (Fig. 5). Regarding the intervention, all six studies performed a HIIT intervention. In all studies, it was measured using MAS. The exercise group was associated with a small improvement in the magnitude of MAS, with a change of 5.38% (ES: 0.048; 95% CI, 0.050 to 0.026; *P* = 0.029; I^2^: 44%) compared to the control group.

Regarding the subgroup analysis considering sex, studies with interventions for boys and girls in the same group did not show a significant change in the magnitude of the MAS of children and adolescents (*n* = 5) (ES: 0.317; 95% CI, -0.013 to 0.647; *P* = 0.060; I^2^: 1%). When boys (*n* = 0) and girls (*n* = 1) were analyzed separately, the analyses were not possible due to the small number of studies. In addition, when analyzing the type of training, all 6 studies performed an intervention with HIIT, and only this method could be analyzed, showing a significant and moderate increase with a change of 5.38% in the magnitude of the MAS in children and adolescents who perform physical exercise (ES: 0.488; 95% CI, 0.050 to 0.926; *P* = 0.02; I^2^: 44%).

Meta-regression analysis of age (β: 0.118; 95% CI; 0.003 to 0.232; *P* = 0.004) and BMI (β: 0.172; 95% CI; 0.052 to 0.292; *P* = 0.005) was associated with an increase in the magnitude of gains in speed. In this way, older children or adolescents are more likely to increase their MAS with physical training than younger children. In relation to BMI, children or adolescents who have a higher BMI are more likely to have increased MAS. However, weekly frequency, duration of session, and follow-up (weeks) were not associated with improvements in this outcome (Table 1).

## Discussion

This meta-analysis examined the effectiveness of different types of exercise training (strength training, aerobic training, HIIT, and sports) on physical fitness components (MF (strength and power); CRF, MAS) of children and adolescents. In addition, we explored the effects of age, sex, BMI, and the principles of physical sports training (volume, intensity, weekly frequency, session duration, intervention duration, and type of physical exercise) on physical fitness trainability. The main results of this study demonstrate that different supervised and structured physical exercises (strength, aerobic, HIIT, and sports training) can significantly improve MF (15–35%), CRF (5.4–8.5%), muscular power (5.6–11.8%), and MAS (5.4%) trainability in children and adolescents. In addition, children and adolescents who perform structured and supervised exercise training present significantly higher values ​​of physical fitness compared to their peers who did not perform any training. Meta-regression analyses identified that MF, CRF, muscular power, and MAS are impacted by physical fitness mediators such as weekly frequency, duration of the session and intervention, and type of training, as well as an individual’s chronological age and BMI.

The evidence presented in the present study can be applied in different contexts, such as disease prevention and treatment, sports training, or school physical education. The prescription of structured physical exercise, which considers the specificities of the pediatric population, is essential for effective exercise training programs in several settings and can guide new research.

### Muscular strength

Our results indicated that boys and girls who practice supervised and structured physical exercises with a focus on strength training, concurrent training (strength and aerobic), or sports participation (ball games or fights) can increase the magnitude of muscular strength (ES: 0.967; 95% CI, 0.635 to 1.299; *P* < 0.001; I^2^: 69%) when compared to individuals who do not practice exercises. However, to optimize gains, sensitivity analyses demonstrate that session duration has an important role. Long-term interventions of strength, concurrent, or sports training seem to have a greater impact on muscular strength gains [45, 53, 106] due to increased muscular stimulation and greater recruitment in the number of muscular fibers and motor units during higher volume training [108]. Tsolaskis et al. (2004) [54] and Lopes et al. (2016) [48] found a high effect size after eight and twelve weeks of strength training and concurrent training for 50–60 min, respectively, increasing the absolute upper- and lower-limb muscular strength of children and adolescents. On the other hand, a small effect size was found after 60 min of sports participation in the Taekwondo class, also increasing lower-limb muscular strength, but in a small proportion when compared with specific strength training [109].

Different types of structured and supervised physical exercise programs produce different magnitudes of muscular strength gains. Our meta-analyses have shown that interventions focusing only on strength training resulted in a large effect size, increasing muscular strength by 31.32%, which is comparable to concurrent training groups (34.49%). These findings show the importance of this type of training for increasing muscle strength. On the other hand, analyses indicate that sports interventions led to a moderate improvement in muscular strength, approximately 15.16%.

For overweight and obese adults, concurrent training and sports such as small-side-game and functional training have been recommended to improve upper and lower body muscular strength. This recommendation stems from the neural adaptations resulting from strength training and the responses induced by exercise, including muscle fiber activation, mitochondrial biogenesis and improved glucose transport [110]. These modalities can be utilized to promote cardiometabolic health in adults with overweight or obesity. On the other hand, our study indicates that for children, strength and concurrent training seem to be the most effective for increasing muscular strength. This difference in effectiveness may be attributed to the fact that children are accustomed to participating in sports from early childhood. Sports activities typically utilize body weight as a resistance, unlike strength or concurrent training, which incorporate additional external loads [46, 102]. Additionally, the specificity of the training performed during assessments and interventions can influence the adaptations. Strength training and concurrent training interventions typically involve exercises such as assessments, whereas sports studies have involved combat training (e.g., Taekwondo) or HIIT (e.g., sprint cycling training) and assessed lower-limb muscular strength using a dynamometer [52, 109]. These adaptations involve changes in agonist-antagonist coactivation, increases in motor unit firing rates, and alterations in the downward drive to motor neurons responsible for muscular contraction [111].

Recent evidence indicates that children and adolescents have low levels of muscular strength [112, 113], which makes the findings of this meta-analysis of great importance for the literature, since increasing muscular fitness (strength, endurance, and muscular power) is associated with better cardiometabolic health in eutrophic, overweight, and obese young individuals during childhood and adult life justified by recent reviews and meta-analyses [4, 28, 114]. In practical applications, several studies have reported interventions for childhood obesity [48, 52, 53, 106, 109]. Lopes et al. (2016) [48] and Shaibi et al. (2006) [53], for example, found that boys and girls can improve muscular strength and insulin sensitivity after concurrent or strength training programs. In this way, Roh et al. (2020) [52] showed that sports, such as taekwondo, can also improve inflammation to provide for obesity and muscular strength. Such improvements are vital for both health and performance. Accordingly, our findings align with guidelines [8, 20] indicating that strength training can be a safe and effective method for enhancing physical fitness in children and adolescents.

### Cardiorespiratory fitness

Our results indicated that supervised and structured physical exercise increased CRF by 6.49% in children and adolescents (ES: 0.400; 95% CI, 0.258 to 0.542; *P* < 0.001; I^2^: 74%). However, the sensitivity analysis demonstrates that the type of training chosen is essential to optimize gains. In this way, it is fully acceptable that studies included in this review that proposed aerobic (ES: 0.514; 95% IC, 0.220 to 1.808; *P* < 0.001; I^2^: 66%), HIIT (ES: 0.668; 95% IC, 0.333 to 1.003; *P* < 0.001; I^2^: 29%) or sports (ES: 0.271; 95% IC, 0.148 to 0.394; *P* < 0.001; I^2^: 15%) presented effects on CRF when compared with only strength training (ES: 0.440; 95% IC, -0.460 to 1.341; *P* = 0.338; I^2^: 93%). In that regard, aerobic exercise and HIIT result in a moderate effect size, increasing by 8.45% and 8.20%, respectively. On the other hand, sports resulted in a small effect size, increasing CRF by 5.40%.

These results can be understood because aerobic fitness reflects the general capacity of the cardiorespiratory system to supply oxygen during sustained physical activity [115]. In this way, exercises of longer duration or intervals, but with high intensity, seem to stimulate greater adaptation in the body through vascular pathways [116]. Although the child has smaller proportions regarding the size of the heart, blood vessels and lungs, the training effects described in our study are explained by the priority of energy demand that children in general have, being susceptible to interval and high intensity activities [23]. However, these benefits can often be attenuated by other conditions, such as overweight. Our results indicated that the effect of physical exercise on CRF was dependent on BMI; that is, the higher the BMI was, the lower the CRF of children and adolescents. These results are in line with other reviews that demonstrate an inverse relationship between these variables [117, 118].

Among the types of exercise aimed at improving CRF and controlling BMI, we highlight aerobic activities, whether HIIT or continuous aerobic training, promote beneficial effects for children. However, for adults, literature highlights that concurrent training, hybrid (sports) and HIIT promote clinically significant changes with an increase of ≥ 3.5 ml•kg^− 1^•min^− 1^ in CRF [110]. Concurrent training, consisting of 176 min per week at moderate intensity, appears to be the most suitable for improving CRF in obese adults, while for children, higher intensity seems to yield better responses. These adaptations result of exercise-induced responses, leading to cardiorespiratory improvements.

The VO_2max_ and the amount of adipose tissue accumulated in the body are both established predictors of CVD, such as hypertension, and are highly correlated with quality of life, morbidity, and mortality [119–121]. CRF has been inversely related to BMI [122, 123], showing that overweight and obese adolescents tend to have a lower CRF and higher risk for cardiometabolic diseases [124]. Recently, Bagatini et al. (2023) [125]. reported that overweight and obese schoolchildren constitute an unfavorable cardiometabolic profile and poor CRF. These results have an important impact according to this meta-analysis, since the importance of carrying out adequate physical training produces significant effects on body fat, justified by a 15-year cohort study conducted by Carnethon et al. (2003) [126], where young adults aged 18 to 30 years with lower CRF levels were more likely to develop diabetes, hypertension, and metabolic syndrome. In this way, this meta-analysis suggests that aerobic, HIIT, and sports training appear to be more effective in increasing CRF trainability than strength and concurrent training for children and adolescents. These types of training should be practiced by children and adolescents to prevent complications associated with obesity and sedentarism and improve cardiometabolic health.

### Muscular power

Our results indicate that physical exercise results in a small improvement of 9.27% in the muscular power of children or adolescents of both sexes when compared to the control group (ES: 0.241; 95% CI, 0.053 to 0.429; *P* = 0.012; I^2^: 0%). These results can be explained by the neural and muscular adaptations that occur with power training, improving the maximum power output in a short time [127]. This answer is related to a greater activation of motor neurons, better activation and synchronization of motor units, improved elastic potential, and greater spinal excitability, which are associated with increased strength and, consequently, muscular power [127]. This is important for sports and health development, influencing physical fitness [4].

Our results indicate that physical exercises (strength training, concurrent training, and HIIT) are safe and can be practiced for children or adolescents, increasing jumping height and muscular power. Recent meta-analyses have indicated that the improvement in MF contributes to a better basal metabolic response and greater muscular metabolic efficiency, improving fat oxidation, glucose uptake and transport due to better insulin sensitivity [86, 128], and this can occur in all age groups, with pre- and postpuberty being more predisposed to improve jumping height in the CMJ test, while mid-puberty has a small effect on trainability [129], which is in accordance with our results that show that children and adolescents can improve their jumping height.

The improvements in MF are indifferent between the frequency of intervention, duration of sessions, or follow-up. This is in line with Markovic et al. (2010) [127], who suggest that short-term intervention (6–15 weeks) can change muscular components and increase muscular power. Arabatzi et al. (2018) [94] showed that a 4-week intervention increased vertical jumping performance in prepubertal children. Similar results were established by Cvetkovic et al. (2018) [97], who showed that a 12-week soccer intervention was able to induce a moderate increase in CMJ in obese children.

Subgroup analysis also showed no difference between the types of exercise, indicating that strength training, concurrent training, or HIIT are responsible for increasing jumping height. This seems to be due to the specificity of activities performed in interventions that require the movement to be performed at high speed and explosiveness [99], using anaerobic metabolism and the stretch-shortening cycle [130] as well as the CMJ test. In soccer, activities such as sprints, jumps, and change of direction result in a moderate effect on muscular power in children [97]. Additionally, strength training and HIIT that involved squats and sprints resulted in an increase in jumping height in the CMJ test and muscular strength [86, 99]. However, no study has analyzed continuous aerobic exercise, presuming that the increase found in CMJ height is a result of the better neural response that anaerobic exercise causes in the neuromuscular system. These results show that all types of exercise should be performed by children and adolescents, so they can benefit from exercise by increasing muscular power.

### Maximum aerobic speed

Children and adolescents who perform supervised and structured physical exercise increased the magnitude of MAS gains when compared to the control group (ES: 0.048; 95% CI, 0.050 to 0.026; *P* = 0.029; I^2^: 44%). These gains are directly linked to the type of training. All studies selected for this meta-analysis analyzed maximal speed from interventions with HIIT, showing that this type of training is efficient in increasing MAS, resulting in a small improvement of 5.38%. These results are related to physiological adaptations induced by training through increased VO_2max_ and running economy [58], resulting from better muscular oxidative capacity and mitochondrial adaptations that occur in HIIT practitioners [131, 132]. Furthermore, executing high intensity running movements requires advanced motor skills [133], as it involves body and neural control for rapid force production, which indicates why adolescents have greater MAS gains than children. Berthoin et al. (1995) [103] found that adolescents of both sexes increased their MAS by approximately 5.5% after a 12-week intervention. These findings are of great relevance since over the years, adolescents tend to reduce the practice of physical exercise, and HIIT is considered a pleasant, dynamic, and short-term activity that can produce important physical fitness gains during the transition from adolescence to adulthood.

MAS gains resulting from training are well established in the literature primarily because HIIT induces a series of physiological adaptations (increases in aerobic and anaerobic metabolism and neuromuscular and cardiovascular functions) [131, 134–136]. However, the transition among childhood, adolescence, and adult life is a period in which, depending on the lifestyle, an excessive accumulation of body fat can occur, developing overweight or obesity and increasing the risks for CVD. In our meta-analyses, most studies analyzed overweight or obese subjects, and we found that subjects with higher BMI have a greater effect on the magnitude of MAS gains, having greater adaptations in response to training, regardless of weekly frequency, session duration, or follow-up duration. Hamila et al. (2017) [71] found in adolescents with a BMI of > 29 kg/m^2^ an increase in the MAS of 8.7%, with a concomitant increase in VO_2max_ of 14.1% after 8 weeks of intervention. In the same way, girls with a BMI of ^33 kg/m2^ increased their MAS from 11.0 to 13.4 km.h^− 1^ after 12 weeks with improvement in insulin sensitivity, an important health marker [107]. On the other hand, children with a BMI of 23.7 kg/m^2^ did not show a significant difference in MAS gains after 6 weeks of training [78]. Thus, obese individuals seem to have a better increase in MAS, improving speed on a larger scale, possibly because they have a larger window of trainability when compared to overweight individuals [137].

Although we found no difference between the training variables, the literature has shown the importance of intensity to improve cardiometabolic health markers [138]. MAS appears to be an important marker for obesity control due to its relationship with CRF and CVD biomarkers. In this way, this review suggests the applicability of this measure through HIIT training to improve cardiometabolic health markers in the pediatric population through pleasant and beneficial training.

### Strengths and methodological limitations

This review presents findings on the highest level of evidence regarding the effects of different physical exercise strategies on the physical fitness trainability of children and adolescents. This systematic review with meta-analysis is the first study to analyze the effects of different supervised and structured physical exercises on physical fitness trainability (MF: muscular strength and muscular power, CRF, and speed) considering chronological age, sex, BMI, and principles of physical sports training in the pediatric population. First, quantifying the effect of the principles of physical sports on the physical fitness trainability of children and adolescents was an important finding of the present study. Second, the benefits found in the analyses have a great social impact, as they indicate that regular physical exercise is important to increase physical fitness levels, resulting in better cardiometabolic health and performance.

However, the present study has some limitations. One of the limitations of this study is related to the number of studies for muscular power and MAS variables, which limited the deeper analysis of different types of training for the pediatric population. Second, although the selected studies had a moderate methodological quality, some studies did not have a description of the training periodization and did not present the necessary data to calculate the volume of exercise, which made the sensitivity analysis of the training variables difficult. Third, the lack of intensity control in the sessions is a general problem since the studies do not propose intensity control and progression during the follow-up. Despite the consensus guidelines that children and adolescents perform activities of moderate-to-vigorous intensity, most of these data are missing, which leaves a gap in the appropriate exercise intensity for this population. Thus, studies of high methodological quality are important and should report the progression of intensity and volume (internal, external, and total load) of exercise sessions to deepen knowledge about training in children and adolescents and estimate the effects of exercise on physical fitness trainability.

## Conclusion

The practice of supervised and structured physical exercise can improve the physical fitness trainability of children and adolescents. In addition, sex and BMI should be considered when prescribing exercise. For the MF, we suggest that boys and girls can be impacted by the increase in strength through all types of exercise. However, activities with muscular strength or concurrent training should be prioritized, as they present greater gains in long-lasting sessions. CRF can be impacted by the practice of supervised physical exercise through activities that require aerobic metabolism, such as aerobic training, sports, or HIIT, and individuals with lower BMI are more impacted by cardiorespiratory gains. To increase muscular power, there is no difference between children and adolescents of both sexes and training variables. Finally, maximal speed is also impacted by physical exercise strategies, and individuals with higher BMI respond better to the intervention, increasing the maximum running speed. Recommendations for future research include carrying out interventions that control the intensity of physical exercise for children and adolescents to improve knowledge about training variables and their relationship with cardiometabolic health and being able to prescribe the ideal dose of physical exercise for each component of physical fitness.

## Electronic supplementary material

Below is the link to the electronic supplementary material.

Supplementary Material 1

Supplementary Material 2

Supplementary Material 3

## Data Availability

The data sets used and/or analyzed during the current systematic review and meta-analysis are included in the review and available from the corresponding author on reasonable request.

## Abbreviations

%BF: Body Fat
Δ%: Percent Change
BMI: Body Mass Index
BORG: Rating of Perceived Exertion
CI: Confidence Interval
CMJ: Countermovement Jump
CRF: Cardiorespiratory Fitness
CVD: Cardiovascular Disease
ES: Effect Size
FFM: Free–Fat Mass
HIIT: High Intensity Interval Training
HR_max_: Maximal Heart Rate
MAS: Maximal aerobic speed
MF: Musculoskeletal Fitness
RM: Repetition Maximal
SD: Standard Deviation
VO_2max_: Maximal oxygen consumption
VO_2peak_: Peak Oxygen Consumption
