# Supplementary material for: Effects of different supervised and structured physical exercise on the physical fitness trainability of children and adolescents: a meta-analysis and meta-regression: Physical fitness trainability in children and adolescents’ health
Source: BMC Pediatr. 2024 Dec 5;24:798. doi: 10.1186/s12887-024-04929-2 (PMC11619429; doi:10.1186/s12887-024-04929-2)
Supplement: Supplementary file 1 — Supplementary Material 1 [file 12887_2024_4929_MOESM1_ESM.pdf]

Effects of different supervised and structured physical exercise on the physical fitness trainability of children and adolescents: a meta-analysis and meta-regression

Physical fitness trainability of children

Supplementary Material

Supplementary Material – 1 – Check list from PRISMA

Reporting checklist for systematic review (with or without a meta-analysis).
Based on the PRISMA guidelines.

		Reporting Item	Page Number
**Title**			
Title	#1	Identify the report as a systematic review	1
**Abstract**			
Abstract	#2	Report an abstract addressing each item in the PRISMA 2020 for Abstracts checklist	3
**Introduction**			
Background/rationale	#3	Describe the rationale for the review in the context of existing knowledge	6-7
Objectives	#4	Provide an explicit statement of the objective(s) or question(s) the review addresses	7
**Methods**			
Eligibility criteria	#5	Specify the inclusion and exclusion criteria for the review and how studies were grouped for the syntheses	8
Information sources	#6	Specify all databases, registers, websites, organisations, reference lists, and other sources searched or consulted to identify studies. Specify the date when each source was last searched or consulted	9
Search strategy	#7	Present the full search strategies for all databases, registers, and websites, including any filters and limits used	9
Selection process	#8	Specify the methods used to decide whether a study met the inclusion criteria of the review, including how many reviewers screened each record and each report retrieved, whether they worked independently, and, if applicable, details of automation tools used in the process	9 e 10
Data collection process	#9	Specify the methods used to collect data from reports, including how many reviewers collected data from each report, whether they worked independently, any processes for	10

		obtaining or confirming data from study investigators, and, if applicable, details of automation tools used in the process	
Data items	#10 a	List and define all outcomes for which data were sought. Specify whether all results that were compatible with each outcome domain in each study were sought (for example, for all measures, time points, analyses), and, if not, the methods used to decide which results to collect	10
Study risk of bias assessment	#11	Specify the methods used to assess risk of bias in the included studies, including details of the tool(s) used, how many reviewers assessed each study and whether they worked independently, and, if applicable, details of automation tools used in the process	10 e 11
Effect measures	#12	Specify for each outcome the effect measure(s) (such as risk ratio, mean difference) used in the synthesis or presentation of results	11
Synthesis methods	#13 a	Describe the processes used to decide which studies were eligible for each synthesis (such as tabulating the study intervention characteristics and comparing against the planned groups for each synthesis (item #5))	11
Synthesis methods	#13 b	Describe any methods required to prepare the data for presentation or synthesis, such as handling of missing summary statistics or data conversions	11
Synthesis methods	#13 c	Describe any methods used to tabulate or visually display results of individual studies and syntheses	11
Synthesis methods	#13 d	Describe any methods used to synthesise results and provide a rationale for the choice(s). If meta-analysis was performed, describe the model(s), method(s) to identify the presence and extent of statistical heterogeneity, and software package(s) used	11
Synthesis methods	#13 e	Describe any methods used to explore possible causes of heterogeneity among study results (such as subgroup analysis, meta-regression)	11
Synthesis methods	#13f	Describe any sensitivity analyses conducted to assess robustness of the synthesised results	11
Reporting bias assessment	#14	Describe any methods used to assess risk of bias due to missing results in a synthesis (arising from reporting biases)	n/a
Certainty assessment	#15	Describe any methods used to assess certainty (or confidence) in the body of evidence for an outcome	11

Data items	#10b	List and define all other variables for which data were sought (such as participant and intervention characteristics, funding sources). Describe any assumptions made about any missing or unclear information	13
**Results**			
Study selection	#16a	Describe the results of the search and selection process, from the number of records identified in the search to the number of studies included in the review, ideally using a flow diagram (http://www.prisma-statement.org/PRISMAStatement/FlowDiagram)	14
Study selection	#16b	Cite studies that might appear to meet the inclusion criteria, but which were excluded, and explain why they were excluded	n/a
Study characteristics	#17	Cite each included study and present its characteristics	14 e 15
Risk of bias in studies	#18	Present assessments of risk of bias for each included study	16
Results of individual studies	#19	For all outcomes, present for each study (a) summary statistics for each group (where appropriate) and (b) an effect estimate and its precision (such as confidence/credible interval), ideally using structured tables or plots	17 a 23
Results of syntheses	#20a	For each synthesis, briefly summarise the characteristics and risk of bias among contributing studies	16
Results of syntheses	#20b	Present results of all statistical syntheses conducted. If meta-analysis was done, present for each the summary estimate and its precision (such as confidence/credible interval) and measures of statistical heterogeneity. If comparing groups, describe the direction of the effect	17 a 23
Results of syntheses	#20c	Present results of all investigations of possible causes of heterogeneity among study results	17 a 23
Results of syntheses	#20d	Present results of all sensitivity analyses conducted to assess the robustness of the synthesised results	17 a 23
Risk of reporting biases in syntheses	#21	Present assessments of risk of bias due to missing results (arising from reporting biases) for each synthesis assessed	n/a
Certainty of evidence	#22	Present assessments of certainty (or confidence) in the body of evidence for each outcome assessed	17 a 23
**Discussion**			
Results in context	#23a	Provide a general interpretation of the results in the context of other evidence	25

Limitations of included studies	#23 b	Discuss any limitations of the evidence included in the review	32
Limitations of the review methods	#23 c	Discuss any limitations of the review processes used	32
Implications	#23 d	Discuss implications of the results for practice, policy, and future research	33
**Other information**			
Registration and protocol	#24 a	Provide registration information for the review, including register name and registration number, or state that the review was not registered	8
Registration and protocol	#24 b	Indicate where the review protocol can be accessed, or state that a protocol was not prepared	8
Registration and protocol	#24 c	Describe and explain any amendments to information provided at registration or in the protocol	10
Support	#25	Describe sources of financial or non-financial support for the review, and the role of the funders or sponsors in the review	34
Competing interests	#26	Declare any competing interests of review authors	34
Availability of data, code, and other materials	#27	Report which of the following are publicly available and where they can be found: template data collection forms; data extracted from included studies; data used for all analyses; analytic code; any other materials used in the review	34

The PRISMA checklist is distributed under the terms of the Creative Commons Attribution License CC-BY. This checklist was completed on 03. March 2023 using https://www.goodreports.org/, a tool made by the EQUATOR Network in collaboration with Penelope.ai
