# Supplementary material for: Effects of different supervised and structured physical exercise on the physical fitness trainability of children and adolescents: a meta-analysis and meta-regression: Physical fitness trainability in children and adolescents’ health
Source: BMC Pediatr. 2024 Dec 5;24:798. doi: 10.1186/s12887-024-04929-2 (PMC11619429; doi:10.1186/s12887-024-04929-2)
Supplement: Supplementary file 2 — Supplementary Material 2 [file 12887_2024_4929_MOESM2_ESM.pdf]

Effects of different supervised and structured physical exercise on the physical fitness trainability of children and adolescents: a meta-analysis and meta-regression

Physical fitness trainability of children

Supplementary Material

Supplementary Material 2 – Boolean search syntax

The following Boolean search syntax was used: ((child OR children OR kid OR teenager OR adolescent OR juvenile OR teens OR youth OR teen) AND ("exercise therapy" OR "resistance training" OR "exercise" OR "aerobic exercise" OR "strength training" OR "physical exercise" OR "Circuit-Based Exercise" OR "Plyometric Exercise" OR "Exercise Movement Techniques" OR "High-Intensity Interval Training" OR "sport*" OR "exercise training" OR "aerobic training" OR "endurance training" OR "weight training")) AND (Musc* OR fitness OR strength OR power OR "motor fitness" OR "motor skill" OR fit* OR "fitness" OR "cardiorespiratory fitness" OR "CRF" OR "strength" OR "muscular strength" OR "agility" OR "velocity" OR "speed" OR "sprint" OR "handgrip"). In addition, the following filters were activated: Clinical Trial and Randomized Controlled Trial. The search included no time or language restrictions. Only eligible full texts in English, Portuguese, or Spanish were considered for review.
