# Supplementary material for: Effects of different supervised and structured physical exercise on the physical fitness trainability of children and adolescents: a meta-analysis and meta-regression: Physical fitness trainability in children and adolescents’ health
Source: BMC Pediatr. 2024 Dec 5;24:798. doi: 10.1186/s12887-024-04929-2 (PMC11619429; doi:10.1186/s12887-024-04929-2)
Supplement: Supplementary file 3 — Supplementary Material 3 [file 12887_2024_4929_MOESM3_ESM.pdf]

Supplementary Material

Supplementary Material – 3 – Risk of bias evaluation

Supplementary table 1. Risk of bias evaluation by TESTEX tool of the **muscular strength** outcome studies.

	Study quality					Study reporting							
**Study**	Eligibility criteria specified	Randomization specified	Allocation concealment	Groups similar at baseline	Blinding of assessor	Outcome measures assessed in 85% of patients	Intention-to-treat analysis	Between-group statistical comparisons reported	Point measures and measures of variability for all reported outcome measures	Activity monitoring in control groups	Relative exercise intensity remained constant	Exercise volume and energy expenditure	**Total Score**
Ben-outhman et al. 2019	1 point	1 point	0 point	1 point	1 point	2 points	0 point	2 points	1 point	0 point	0 point	1 point	Medium quality
Benson et al. 2008	1 point	1 point	1 point	1 point	1 point	3 points	1 point	2 points	1 point	0 point	1 point	1 point	High quality
Brandão et al. 2015	1 point	1 point	0 point	1 point	0 point	2 points	0 point	1 point	1 point	0 point	1 point	1 point	Medium quality
Chuensiri et al. 2017	1 point	1 point	0 point	1 point	0 point	1 point	0 point	2 points	1 point	0 point	1 point	1 point	Medium quality
Cohen et al. 2021	1 point	0 point	1 point	1 point	0 point	2 points	0 point	2 points	1 point	1 point	1 point	1 point	High quality
Faigembaum et al. 1993	1 point	0 point	0 point	1 point	0 point	2 points	0 point	1 point	1 point	0 point	1 point	1 point	Medium quality
Faigembaum et al. 2002	0 point	0 point	0 point	1 point	0 point	3 points	0 point	2 points	1 point	0 point	1 point	1 point	Medium quality
Ingle et al. 2006	1 point	1 point	0 point	0 point	0 point	1 point	0 point	2 points	1 point	0 point	1 point	1 point	Medium quality
Kim et al. 2011	1 point	1 point	0 point	0 point	0 point	0 point	0 point	0 point	1 point	0 point	0 point	1 point	Low quality
Lopes et al. 2016	1 point	0 point	0 point	0 point	0 point	2 points	0 point	2 points	0 point	0 point	1 point	1 point	Medium quality
Lubans et al. 2010	1 point	1 point	0 point	1 point	0 point	2 points	1 point	2 points	0 point	0 point	1 point	1 point	Medium quality
Ozmum et al. 1994	0 point	0 point	0 point	1 point	0 point	1 point	0 point	1 point	0 point	0 point	0 point	0 point	Low quality
Ransay et al. 1990	0 point	0 point	0 point	0 point	0 point	0 point	0 point	1 point	0 point	0 point	1 point	1 point	Low quality
Roh et al. 2020	1 point	1 point	0 point	1 point	0 point	0 point	0 point	2 points	1 point	1 point	0 point	0 point	Medium quality
Shaibi et al. 2006	1 point	1 point	0 point	1 point	0 point	2 points	0 point	2 points	1 point	1 point	1 point	1 point	High quality
Tsolakis et al. 2004	0 point	0 point	0 point	1 point	0 point	1 point	0 point	2 points	0 point	0 point	0 point	1 point	Low quality

0 point | 1 point | 2 points | 3 points

Low quality | Medium quality | High quality

Supplementary table 2. Risk of bias evaluation by TESTEX tool of the **VO₂** outcome studies.

	Study quality					Study reporting							
**Study**	Eligibility criteria specified	Randomization specified	Allocation concealment	Groups similar at baseline	Blinding of assessor	Outcome measures assessed in 85% of patients	Intention-to-treat analysis	Between-group statistical comparisons reported	Point measures and measures of variability for all reported outcome measures	Activity monitoring in control groups	Relative exercise intensity remained constant	Exercise volume and energy expenditure	**Total Score**
Almeida_ et al. 2020													
Alves et al. 2015													
Alves et al. 2016													
Almeida et al. 2020													
Baquet et al. 2002													
Baquet_et al. 2010													
Barbeau et al. 2007													
BenOunis et al. 2010													
Benson et al. 2008													
Berntsen et al. 2010													
Brandao et al. 2015													
Carrel et al. 2005													
Chae et al. 2010													
Chuensiri et al. 2018													
Crouter et al. 2015													
Davi et al. 2012													
Davis et al. 2020													
Fairclough et al. 2016													
Faude et al. 2010													
Garcia_Hermoso et al. 2020													
Hamila et al. 2017													
Harrel et al. 1996													
Hergenroeder et al. 1993													
Ignico et al. 1995													

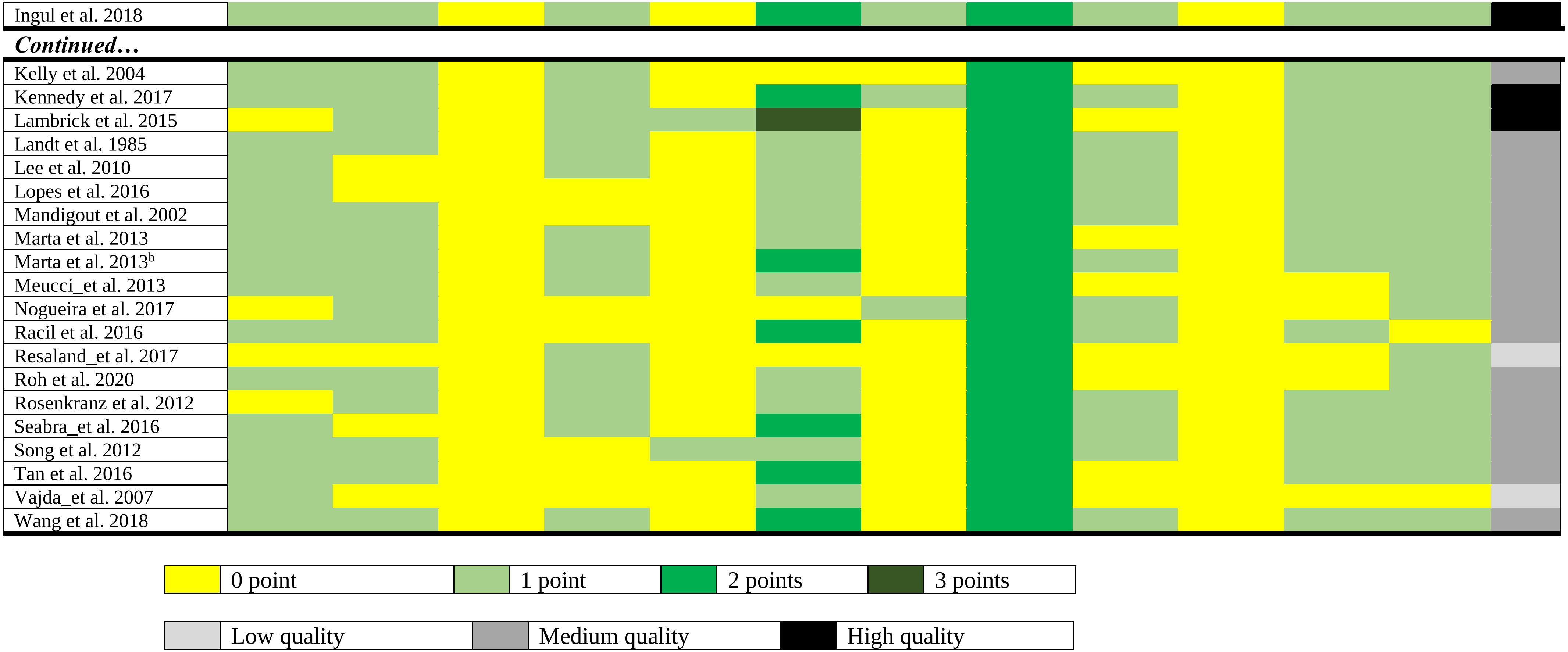
Ingul et al. 2018
Continued…
Kelly et al. 2004
Kennedy et al. 2017
Lambrick et al. 2015
Landt et al. 1985
Lee et al. 2010
Lopes et al. 2016
Mandigout et al. 2002
Marta et al. 2013
Marta et al. 2013[b]
Meucci_et al. 2013
Nogueira et al. 2017
Racil et al. 2016
Resaland_et al. 2017
Roh et al. 2020
Rosenkranz et al. 2012
Seabra_et al. 2016
Song et al. 2012
Tan et al. 2016
Vajda_et al. 2007
Wang et al. 2018
0 point
1 point
2 points
3 points
Low quality
Medium quality
High quality

Supplementary table 3. Risk of bias evaluation by TESTEX tool of the **Counter Movement Jump** outcome studies.

	**Study quality**					**Study reporting**							
**Study**	Eligibility criteria specified	Randomization specified	Allocation concealment	Groups similar at baseline	Blinding of assessor	Outcome measures assessed in 85% of patients	Intention-to-treat analysis	Between-group statistical comparisons reported	Point measures and measures of variability for all reported outcome measures	Activity monitoring in control groups	Relative exercise intensity remained constant	Exercise volume and energy expenditure	**Total Score**
Alves_et al. 2015	1 point	1 point	0 point	1 point	0 point	2 points	0 point	2 points	1 point	0 point	1 point	1 point	Medium quality
Arabatzi 2017	0 point	1 point	0 point	1 point	0 point	2 points	0 point	2 points	0 point	0 point	0 point	0 point	Medium quality
Bem Othman et al. 2019	0 point	1 point	0 point	1 point	1 point	3 points	0 point	2 points	1 point	0 point	1 point	1 point	High quality
Bogotaj et al. 2021	1 point	1 point	0 point	0 point	0 point	3 points	0 point	2 points	1 point	0 point	1 point	1 point	Medium quality
Buchan et al. 2012	0 point	1 point	0 point	1 point	0 point	1 point	0 point	2 points	1 point	0 point	1 point	1 point	Medium quality
Cvetkovic et al. 2018	1 point	1 point	0 point	0 point	1 point	3 points	0 point	2 points	1 point	0 point	1 point	1 point	High quality
Granacher et al. 2011	0 point	1 point	0 point	1 point	0 point	2 points	0 point	2 points	1 point	1 point	1 point	1 point	Medium quality
Granacher et al. 2011	0 point	1 point	0 point	1 point	0 point	2 points	0 point	2 points	1 point	0 point	1 point	1 point	Medium quality
Marta et al. 2013	1 point	1 point	0 point	1 point	0 point	2 points	0 point	2 points	1 point	1 point	1 point	1 point	High quality
Marta_et al. 2019	1 point	1 point	0 point	1 point	0 point	2 points	0 point	2 points	1 point	0 point	1 point	1 point	Medium quality
Muelhbauer et al. 2012	0 point	1 point	0 point	1 point	0 point	3 points	0 point	2 points	1 point	0 point	1 point	1 point	Medium quality
Racil et al. 2016	1 point	0 point	0 point	0 point	0 point	2 points	0 point	2 points	1 point	0 point	1 point	0 point	Medium quality
Racil_ et al. 2017	0 point	0 point	0 point	1 point	0 point	2 points	0 point	2 points	1 point	0 point	0 point	1 point	Medium quality

0 point | 1 point | 2 points | 3 points

Low quality | Medium quality | High quality

Supplementary table 4. Risk of bias evaluation by TESTEX tool of the **Maximal Aerobic Speed** outcome studies.

	Study quality					Study reporting							
**Study**	Eligibility criteria specified	Randomization specified	Allocation concealment	Groups similar at baseline	Blinding of assessor	Outcome measures assessed in 85% of patients	Intention-to-treat analysis	Between-group statistical comparisons reported	Point measures and measures of variability for all reported outcome measures	Activity monitoring in control groups	Relative exercise intensity remained constant	Exercise volume and energy expenditure	**Total Score**
**Sprint**													
Abassi et al. 2020	1	1	0	1	0	1	0	2	1	0	1	1	Medium quality
Baquet et al. 2002	0	0	0	1	0	2	0	2	1	0	1	1	Medium quality
Berthoin et al. 1995	0	0	0	0	0	1	0	2	1	0	1	1	Medium quality
Hamila et al. 2017	1	1	0	1	0	2	0	2	1	0	1	1	Medium quality
Lambrick et al. 2015	1	1	0	1	0	3	0	2	0	0	1	1	Medium quality
Lau et al. 2014	1	0	0	0	0	1	0	2	1	0	1	1	Medium quality

Legend: 0 point | 1 point | 2 points | 3 points

Low quality | Medium quality | High quality
